# Associations between Glyphosate Exposure and Glycemic Disorders: A Focus on the Modifying Effect of Sex Hormones

**DOI:** 10.3390/toxics12080600

**Published:** 2024-08-18

**Authors:** Yu Dong, Yuan Li, Liwen Ma, Shuge Shu, Jiawen Ren, Xiangyu Yu, Dan Luo, Zhizhou Duan, Yongquan Yu

**Affiliations:** 1 Key Laboratory of Environmental Medicine Engineering, Ministry of Education, School of Public Health, Southeast University, Nanjing 210000, China; dong_yu@seu.edu.cn (Y.D.); ssg03300420@163.com (S.S.); rjw67516@163.com (J.R.); yxy000923@163.com (X.Y.); 2 Department of Cosmetic Dermatology, The Fifth People’s Hospital of Hainan Province, Haikou 570000, China; liyuanskin@hotmail.com; 3 Department of Dermatology, The First Affiliated Hospital of Nanjing Medical University, Nanjing 210000, China; maliwents@163.com (L.M.); daniluo2005@163.com (D.L.); 4 Preventive Health Service, Jiangxi Provincial People’s Hospital, The First Affiliated Hospital of Nanchang Medical College, Nanchang 330000, China

**Keywords:** glyphosate, glucose homeostasis, sex hormone-binding globulin, interaction

## Abstract

Widespread glyphosate contamination in the environment and its endocrine-disrupting potential are concerning. However, evidence of glyphosate’s effects on glycemic health is limited. To examine the association between glyphosate and glucose homeostasis in the general US population, a total of 3038 individuals were enrolled from the 2013-2016 cycles of the National Health and Nutrition Examination Survey (NHANES). Survey-weighted linear regression and restricted cubic spline curves were used to detect the associations between glyphosate and glycemic disorders. The effects of interactions between sex hormones and glyphosate on glycemic outcomes were evaluated. The results showed that glyphosate was significantly linked to increased glycated hemoglobin A1c (HbA1c) levels (*β* = 0.01; 95%CI, 0.01 to 0.02; *p* = 0.001) and the compromised homeostatic model assessment of beta-cell function (HOMA-beta) scores (*β* = −0.09; 95%CI, −0.17 to −0.01; *p* = 0.024). More importantly, these “glyphosate–glycemic disorder” associations were significantly modified by sex hormone-binding globulin (SHBG; *P* for interaction < 0.05), with more pronounced relationships being identified in individuals with low SHBG levels. Our findings indicate that glyphosate is correlated with glucose dyshomeostasis. Individuals with low SHBG levels exhibited susceptibility to glyphosate-related glycemic toxicity; therefore, it might be prudent to determine glycemic health in those subjects with glyphosate exposure.

## 1. Introduction

Glyphosate, or N-(phosphonomethyl) glycine, is a post-emergent and nonvolatile organophosphorus agricultural compound. It is commonly used as the primary ingredient in glyphosate-based herbicides, such as Roundup [1,2]. Since its commercialization in the 1970s, glyphosate has become one of the best-selling agricultural pesticides worldwide, as it has effective and non-selective systemic herbicidal activity and has a low production cost [3]. Notably, with the widespread use of genetically modified glyphosate-resistant crop variants worldwide, the application volumes of glyphosate have increased dramatically [4]. In 2015, the annual use of glyphosate worldwide was estimated to be from 600 thousand to 750 thousand metric tons, and this was expected to increase to 740 thousand–920 thousand metric tons by 2025 [5]. As the application volumes of glyphosate increase, it is important to note that the high environmental dissemination of glyphosate may cause serious residual problems. Glyphosate residues have been frequently detected in soil, water, air, food, and even human samples [6]. It has been reported that the concentrations of urinary glyphosate varied from 1.54 to 434.73 nM in occupationally exposed subjects, while in individuals from the general population, the urinary glyphosate levels ranged from 1.54 to 44.95 nM [7]. In addition, a positive temporal trend was observed in a proportion of subjects who had detectable levels of urinary glyphosate in California from 1993 (12%) to 2016 (70%) [8]. Studies also found widespread glyphosate contamination in several European countries, and the detectable rate of glyphosate in children ranged between 17% and 60% [9,10]. In a recent study, using data from the 2013–2014 cycle of the National Health and Nutrition Examination Survey (NHANES), the authors also found that over 75% of the studied individuals in the United States (US) had detectable levels of glyphosate in their urine, suggesting that humans are subject to widespread and rising exposure to glyphosate [11].

Glyphosate kills plants by targeting and affecting chloroplastic 5-enolpyruvylshikimate-3-phosphate synthase, an essential plant-specific enzyme that mammals are deficient in. Therefore, it was long believed to be the “least toxic” chemical for humans [12]. However, recently, public concerns regarding the potential detrimental effects of glyphosate on human health have been raised, as numerous independent researchers have revealed the associations between glyphosate exposure and several cancer and non-cancer health dysfunctions [13,14,15]. Diabetes is one of the fastest-growing metabolic diseases worldwide. Based on the diabetes map from the International Diabetes Federation, more than 537 million individuals had diabetes in 2021, and this number is expected to increase to 783 million by 2025 [16]. It is estimated that 6.7 million individuals died from diabetes-associated complications in 2021, contributing to 12.2% of deaths worldwide [17]. Diabetes also poses threats to communities’ economies and is associated with several social problems, such as the significant consumption of healthcare resources and reduced work productivity [18]. Dysregulation of glucose homeostasis means a risk of developing diabetes. Therefore, the need for more research on the modifiable environmental risk factors for glycemic disorders is becoming urgent. Experiments on animals have indicated that glyphosate dose-dependently increased fasting plasma glucose (FPG) and serum insulin concentrations, compromised insulin function, and induced glucose intolerance via NFκB-mediated hepatic inflammation in rats [19]. Combined exposure to a high-fructose diet and glyphosate significantly increased the levels of fasting blood glucose, insulin, triglycerides, and uric acid in Wistar rats [20], suggesting that glyphosate is a widely distributed pollutant with glucose homeostasis-interfering potential. However, these studies mainly focused on the deleterious effects of exposure in laboratory animals, and epidemical evidence of the association between glyphosate and glucose homeostasis is rather limited.

Sex hormones are naturally occurring compounds that could be influenced by environmental endocrine-disrupting chemicals (EDCs) [21,22]. These signaling hormones are involved in the regulation of glucose homeostasis [23]. For instance, in a case–control study in rural China, both independent and combined neonicotinoid pesticide exposures were significantly correlated with an increased risk of diabetes in females, and serum testosterone (T) was demonstrated to be potential mediator in theses associations [24]. In addition, serum T mediated the association between organochlorine pesticides and diabetes in males, with the corresponding proportion being 21.149% [25]. 17β-estradiol (E2) was shown to attenuate streptozotocin-induced hyperglycemia and improve the pancreatic functions in diabetic mice [26]. Sex hormone-binding globulin (SHBG) is a liver-synthesized glycated homodimeric protein that regulates circulating sex hormone levels via the binding and transport of free T and E2 [27]. Previous clinical studies have indicated that low serum SHBG levels are associated with impaired glucose homeostasis and the increased risk of diabetes [28]. Moreover, several single-nucleotide polymorphisms in the SHBG gene, which might subsequently lead to alterations in serum SHBG levels, were shown to be related to higher risks of insulin resistance [29]. Associations between glyphosate and sex hormones have also been reported [30]. Nonetheless, it is unclear whether sex hormones play roles in glyphosate-related glucose homeostasis disruption.

Therefore, in the present study, we analyzed representative data from cross-sectional National Health and Nutrition Examination Survey (NHANES) samples in the United States. Individuals who participated in the 2013–2016 survey cycles were enrolled, as they are associated with complete data on glyphosate exposure, glycemic outcomes, and sex hormones simultaneously. We used six parameters from glucose homeostasis tests for the evaluation of glycemic health, namely FPG, fasting insulin (FINS), glycated hemoglobin A1c (HbA1c), homeostasis model assessment of insulin resistance (HOMA-IR), homeostasis model assessment of insulin sensitivity (HOMA-IS), and homeostatic model assessment of beta-cell function (HOMA-beta). We fitted survey-weighted linear regression models to determine the associations between urinary glyphosate exposure and glucose homeostasis. We also examined possible effect modifiers between the above-mentioned associations. Considering the endocrine-disrupting properties of glyphosate, we mainly focused on the effect of interaction between sex hormones and glyphosate on glycemic outcomes to determine whether sex hormones modify the association between environmental glyphosate exposure and glycemic disorders.

## 2. Materials and Methods

### 2.1. Study Population

The NHANES refers to a cross-sectional and multistage designed survey that is performed to evaluate the health and nutritional status of a representative civilian populations in the United States. This survey is implanted periodically by the National Center for Health Statistics (NCHS) and contains a wide range of laboratory and questionnaire information, including basic demographic data, health status, and environmental-toxicant exposure. The study protocol of the NHANES was reviewed and approved by the NCHS Research Ethics Review Board. Informed consent was provided by all included subjects prior to participation, and none of the authors in this study was involved in the production of the NHANES.

In the present study, we collected and analyzed data from the NHANES 2013–2016 cycles in which urinary glyphosate, serum sex hormones, and glycemic outcomes were evaluated simultaneously. Initially, a total of 11,329 participants aged 20 years or above were included. Then, participants with missing data on glyphosate exposure were subsequently excluded (*n* = 8261). After further excluding participants with missing data on glycemic outcomes, the final study identified 1428 individuals for FPG analysis, 2995 for HbA1c analysis, 1403 for FINS analysis, and 1400 for HOMA parameter analyses. A detailed overview of the screening process is shown in the flowchart in Supplementary Figure S1.

### 2.2. Exposure Measurement

Individuals’ exposure to glyphosate was determined based on the urinary concentration of glyphosate. The main transformation product of glyphosate, aminomethylphosphonic acid (AMPA), was not included in this study because it was not measured in the NHANES database. Briefly, urine samples were collected from each participant in mobile examination centers using sterile urine-sample collectors, and they were stored at the recommended temperatures until analysis took place. The highly selective analytical method 2D-on-line ion chromatography–tandem mass spectrometry (IC-MS/MS), coupled with isotope dilution quantification, was applied to evaluate the concentrations of urinary glyphosate. The values of glyphosate were expressed as ng/mL, and the lower limit of detection (LLOD) was 0.200 ng/mL. Undetected glyphosate (below the LLOD) was calculated as the LLOD divided by the square root of 2 based on the NHANES analysis guidelines. Detailed information regarding the qualification of urinary glyphosate is available from the laboratory methods section of the NHANES website (https://wwwn.cdc.gov/nchs/nhanes/Default.aspx (accessed on 2 June 2024)).

### 2.3. Outcomes Ascertainment

A total of 6 parameters of glucose homeostasis were selected: FPG, FINS, HbA1c, HOMA-IR, HOMA-IS, and HOMA-beta. Of these, FPG, FINS, and HbA1c were measured using venous blood samples of the enrolled individuals, which were collected after they had fasted for at least 8 h. Serum FPG was quantified via the hexokinase method using a UV-based Roche/Hitachi Cobas C Chemistry Analyzer. A two-site immune-enzymometric assay was performed to detect FINS using a Tosoh AIA System analyzer. The levels of HbA1c in whole-blood specimens were evaluated using a Tosoh G8 Glycohemoglobin Analyzer. HOMA-IR, HOMA-IS, and HOMA-beta were calculated based on the following formulas:

HOMA-IR = [FPG (mmol/L) × FINS (μU/mL)]/22.5

HOMA-IS = 1/HOMA-IR

HOMA-beta = 20 × FINS (μU/mL)/[FPG (mmol/L) − 3.5]

Detailed information regarding the qualification of parameters of glucose homeostasis is available from the laboratory methods section of the NHANES website (https://wwwn.cdc.gov/nchs/nhanes/Default.aspx (accessed on 2 June 2024)).

### 2.4. Sex Hormones’ Measurement

Three main sex hormones in the human body were selected as potential modifiers: serum total T (TT), E2, and SHBG. Of them, the serum levels of TT and E2 were detected using isotope dilution–high-performance liquid chromatography–tandem mass spectrometry (ID-LC–MS/MS). The serum levels of SHBG were quantified based on the measurement of the corresponding immuno-antibodies and chemo-luminescence reaction products. Undetected sex hormones (below the LLOD) were calculated as the LLOD divided by the square root of 2 based on the NHANES analysis guidelines. Detailed information regarding the qualification of serum sex hormones is available from the laboratory methods section of the NHANES website (https://wwwn.cdc.gov/nchs/nhanes/Default.aspx (accessed on 2 June 2024)).

### 2.5. Covariates

Demographic data (age, gender, ethnicity, educational level, and family-income-to-poverty ratio (PIR)), lifestyle data (smoking status, alcohol consumption, and physical activity), body mass index (BMI), medical history of cardiovascular disease (CVD) and stroke, and urinary creatinine levels were selected as covariates based on a prior empirical study [31]. The latent correlations among these covariates, glyphosate exposure, and glycemic outcomes are illustrated in a directed acyclic graph (DAG) in Supplementary Figure S2.

Individuals’ PIRs were stratified into two groups, <1 and ≥1, and BMI was grouped into three grades: < 25kg/m^2^, 25–30 kg/m^2^, and ≥30 kg/m^2^. Physical activity status was determined according to the participants’ responses to the self-report questions and was categorized into three levels: no physical activity, moderate physical activity, or vigorous physical activity. Each subject’s exposure to cigarettes was determined based on the concentrations of serum cotinine, a major metabolite of nicotine that was widely used as a suitable internal exposure marker of cigarettes [32]. The cutoff value of cotinine was set at 0.015 ng/mL (LLOD), based on the prior studies [33,34]. Individuals with serum cotinine ≥ the LLOD (0.015 ng/mL) were identified as cigarette-exposed populations, including active tobacco smoking, e-cigarette smoking, and passive cigarette smoking. Participants’ drinking status was evaluated according to the statement, “had at least 12 alcohol drinks per year”, and subjects who responded with the affirmative were defined as drinkers. Medical history of CVD was determined according to the responses to the question: “ever told you had a congestive heart failure, coronary heart disease or angina”.

### 2.6. Statistical Analysis

The continuous variables of age, creatinine levels, sex hormone levels and glyphosate levels were expressed as means [±standard deviation (SD)], while other categorical variables were expressed as frequencies [percentage (%)]. As the levels of urinary glyphosate, serum sex hormones, and glucose homeostasis parameters were in right-skewed distributions (*p* < 0.05 for all, Kolmogorov–Smirnov test), they were natural logarithm-transformed to improve the normality in the subsequent analyses.

Linear regression was performed to explore the associations between glyphosate exposure and continuous outcomes (FPG, FINS, HbA1c, HOMA-IR, HOMA-IS, and HOMA-beta), and the estimated results were expressed as coefficients (*β*) with corresponding 95% confidence intervals (CIs). Three sequential analytic models were included: Model 1 was univariate linear regression. Model 2 was multivariate linear regression controlled for urinary creatinine and basic demographic covariates, including age, gender, ethnicity, PIR, and educational level. Model 3 was further adjusted for obesity, alcohol consumption, physical activity, cigarette exposure, stroke, and CVD based on model 2. In addition, the complex design of the NHANES and subsample weight were considered by including the variables stratum, cluster, and sample weight (WTSSCH2Y and WTSSGL2Y, computed by dividing the 2-year glyphosate surplus specimen 2-year weights C by 2), based on the NHANES guidelines. Missing values of covariates (*n* = 355 for FPG, *n* = 338 for FINS, *n* = 749 for HbA1c and *n* = 338 for HOMA parameters) were not deleted in this study; instead, they were calculated using multiple imputation by chained equations. We created ten imputed datasets to allow for the variance of imputation, and the final estimates were pooled and obtained from the results in each dataset, using Rubin’s rules. Three analyses were performed to determine the robustness of the regression models: (1) all data were reanalyzed without considering the NHANES survey design and the subsample weights of glyphosate; (2) a strategy of case-wise deletion for all missing data was conducted, and the associations between glyphosate exposure and glycemic disorders were reanalyzed; and (3) we calculated the false discovery rate (FDR) as *P*_FDR_ value of significant results to account for multiple comparisons.

The multiplicative interaction effects of potential modifiers and urinary glyphosate on the above associations were examined using a likelihood ratio test. Model formula:

$$Y=\beta +\beta_{Covariate}X_{Covariate}+\beta_{SHBG}X_{SHBG}+\beta_{Glyphosate}X_{Glyphosate}+\beta_{SHBG*Glyphosate}X_{SHBG}X_{Glyphosate}$$

where *β*, *β_covariate_*, *β_SHBG_*, *β_Glyphosate_*, and β_SHBG*Glyphosate_ are the coefficients of constant, covariate, SHBG, glyphosate, and interaction, respectively. The joint effects of selected modifiers with statistical significance and glyphosate on glycemic outcomes were assessed by categorizing individuals into three groups, based on their median levels: (1) high-SHBG + low-glyphosate exposure group, (2) low-SHBG + low-glyphosate/high-SHBG + high-glyphosate exposure group, and (3) low-SHBG + high-glyphosate exposure group. The high-SHBG and low-glyphosate group was set as the reference group with low risk of glycemic disorders.

In addition, considering that the estimates of linear regression were calculated based on the assumption that exposure and outcomes were linearly related, we further conducted restricted cubic spline (RCS) analysis with three knots (10th, 50th, and 90th percentiles) to determine the nonlinear association between natural log-transformed glyphosate levels and parameters of glucose homeostasis, as well as to graphically characterize the potential dose–effect relationships. The violation of linearity was examined using the Wald test. Segmented regression was constructed to fit the piecewise-linear relationships between glyphosate and glycemic outcomes if nonlinearity was observed. The threshold level of urinary glyphosate was determined once a recursive algorithm further provided the optimal inflection point. The interaction curves of selected modifiers were also depicted to visualize the interaction effect.

All data were analyzed with R software, using “survey”, “rms”, “dplyr”, “caret”, and “mice” packages (version 4.1.2). Estimates were significant when *p*-values ≤ 0.05 (two-sided).

## 3. Results

### 3.1. Descriptive Statistics of the Study Subjects

Table 1 displays the demographic characteristics of the participants examined in the 2013–2016 survey cycles of the NHANES. Among the representative participants included in the HOMA parameters’ analysis, the mean age was 50.19 years, and females accounted for 52.9% of the group, respectively. The majority of subjects were non-Hispanic White individuals (42.5%), obese (BMI ≥ 30 kg/m^2^, 40.1%), and at or above the poverty level (78.2%). Approximately 32.2% of individuals received some college or AA degree education. More than 50% of subjects were perceived to have the conditions of alcohol consumption, cigarette exposure (including active tobacco smoking, e-cigarette smoking, and passive cigarette smoking), and physical inactivity. A small proportion of individuals was identified to have a history of CVD (11.1%) and/or stroke (3.5%). The average level of glyphosate was around 0.489 ng/mL, and the detectable rate was 71.89%. The mean concentrations and the detectable rate of urinary creatinine, serum TT, E2, and SHBG were around 122.55 mg/100 mL (100%), 225.08 ng/dL (100%), 64.17 pg/mL (94.83%), and 63.78 nmol/L (100%), respectively. Similar distribution patterns of demographic characteristics were identified for participants included in the FPG, FINS, and HbA1c analyses.

### 3.2. Associations between Urinary Glyphosate and Glycemic Outcomes

As shown in Table 2 and Supplementary Table S1, consistent and significant associations were identified between urinary glyphosate exposure and the increased risk of compromised glycemic HOMA-beta and HbA1c parameters across the three models. Thus, the model adjusting for all covariates (model 3) was used in the subsequent analyses. In participants enrolled for analyses of glycemic parameters, ln-transformed urinary glyphosate was negatively associated with HOMA-beta (*β* = −0.09, 95% CI: −0.17 to −0.01, *p* = 0.024, *P*_FDR_ = 0.042) and positively associated with HbA1c (*β* = 0.01, 95% CI: 0.01 to 0.02, *p* = 0.001, *P*_FDR_ = 0.042). All the significant results in Table 2 had a *P*_FDR_ value < 0.05. As shown in Supplementary Tables S2 and S3, the significant associations of glyphosate with HbA1c and HOMA-beta remained consistent in unweighted analyses and case-wise deletion analyses.

As depicted in Supplementary Figure S3, no significant violation of linearity was identified in the associations of ln-transformed urinary glyphosate with FPG, FINS, HOMA-beta, HOMA-IR, and HOMA-IS (*p* > 0.05). Similar to the results of linear regressions, the RCS curves indicated sharp and negative linear associations between glyphosate and HOMA-beta. Meanwhile, the associations of glyphosate with HbA1c were nonlinear (*p* < 0.05), and the restricted cubic plots were inverted U-shaped curves. Segmented regressions indicated that the threshold of glyphosate was −0.689 (0.502 ng/mL) for HbA1c. To the left of the threshold points, the risk of HbA1c levels increased drastically as the levels of glyphosate increased. No significant nonlinear correlation between glyphosate and other outcomes was observed.

### 3.3. Effect of Low Circulating SHBG on Association of Urinary Glyphosate with Glycemic Outcomes

The results of the interaction effect analyses (Supplementary Table S4) indicated that gender, ethnicity, educational level, smoking status, alcohol consumption, physical activity, body measure index (BMI), serum TT, and serum E2 did not modify the associations of urinary glyphosate with glycemic variables (*P* for interaction > 0.05); the only marginally significant interactive relationships identified were between SHBG and glyphosate on HOMA-IR, HOMA-IS, and FINS (*P* for interaction = 0.023, 0.023, and 0.024, respectively) (*P*_FDR_ for interaction = 0.051, 0.051, and 0.051, respectively) (Table 2). Additionally, significant associations were identified between SHBG and compromised glycemic parameters HOMA-IR, HOMA-IS, FINS, HbA1c, and FPG parameters (*P*_FDR_ < 0.001, <0.001, <0.001, <0.001, and <0.001, respectively) (Table 2). Therefore, SHBG was selected as a potential modifier in the association between glyphosate and glycemic outcomes. As shown in Table 3, significant associations of glyphosate with HOMA-IR (*β* = −0.13, 95% CI: −0.25 to −0.02, *p* = 0.024, *P*_FDR_ = 0.056), HOMA-IS (*β* = 0.13, 95% CI: 0.02 to 0.25, *p* = 0.024, *P*_FDR_ = 0.056), and FINS (*β* = −0.14, 95% CI: −0.24 to −0.03, *p* = 0.013, *P*_FDR_ = 0.056) were found in individuals with high circulating SHBG concentrations.

**Figure 1:**
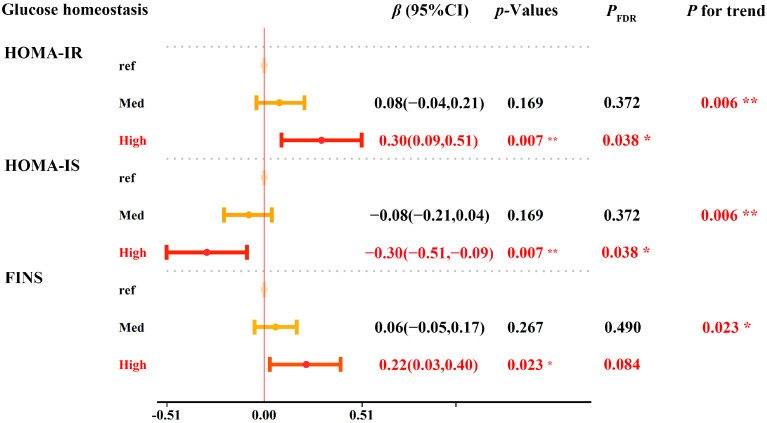
Joint effects between glyphosate exposure and SHBG on glycemic disorder. Participants were categorized into three groups based on their median levels of glyphosate and SHBG. Group 1 was the high-SHBG + low-glyphosate group, which was set as the reference group. Group 2 was the low-SHBG + low-glyphosate/high-SHBG + high-glyphosate group, which was considered as the medium risk group. Group 3 was the low-SHBG + high-glyphosate group, which was considered as the high-risk group. The models were adjusted by age, gender, ethnicity, educational level, family-income-to-poverty ratio, cigarette exposure, alcohol consumption, physical activity, body mass index, cardiovascular disease, stroke, and urinary creatinine levels. Abbreviations: CI, confidence interval; SHBG, sex hormone-binding globulin; HOMA-IR, homeostasis model assessment of insulin resistance; HOMA-IS, homeostasis model assessment of insulin sensitivity; FINS, fasting insulin. * *p* < 0.05; ** *p* < 0.01.

Moreover, the interaction curve demonstrated that serum SHBG markedly alleviated the glyphosate-related increase in the risk of HOMA-IR and FINS, while it increased the glyphosate-related increase in HOMA-IS (Supplementary Figure S4A–C). In addition, the restricted cubic plot showed linear and positive relationships of urinary glyphosate with HOMA-IR and FINS, and a linear and negative relationship with HOMA-IS was shown in individuals with low SHBG levels, but not in the high SHBG group (Supplementary Figure S4D–F). The combined effects of SHBG and glyphosate on HOMA-IR, HOMA-IS, and FINS are illustrated in Figure 1. Compared with the reference group (high-SHBG + low-glyphosate group), participants in the low-SHBG + high-glyphosate group had increased HOMA-IR (*β* = 0.30, 95%CI: 0.09 to 0.51, *p* = 0.007, *P*_FDR_ = 0.038, *P* for trend = 0.006), decreased HOMA-IS (*β* = −0.30, 95%CI: −0.51 to −0.09, *P* = 0.007, *P*_FDR_ = 0.038, *P* for trend =0.006) and increased FINS levels (*β* = 0.22, 95% CI: 0.03 to 0.40, *p* = 0.023, *P*_FDR_ = 0.084, *P* for trend = 0.023). Most of the significant results in Figure 1 have a *P*_FDR_ value < 0.05. As shown in Supplementary Table S5, no significant result of the mediation analysis was identified for SHBG on the association between glyphosate and glycemic outcomes, excluding the possibility that SHBG could act as mediating variable in the association between glyphosate and glycemic disorders.

## 4. Discussion

In this study, based on the data obtained from the 2013–2016 cycles of the NHANES, we found that the natural logarithm-transformed urinary glyphosate was significantly and positively associated with HbA1c levels, and it was simultaneously negatively associated with HOMA-beta scores in the general adults of the US. In addition, SHBG was the only modifier with statistical significance in the association of glyphosate with HOMA-IR, HOMA-IS, and FINS, and the detrimental relationships between glyphosate, and these outcomes were more apparent in subjects with low circulating SHBG levels. The interaction curve further demonstrated that low SHBG markedly aggravated the glyphosate-associated increase in HOMA-IR and FINS and the decrease in HOMA-IS, suggesting that participants with low serum SHBG concentrations exhibited susceptibility to glyphosate-related glycemic toxicity. Our study constitutes the first investigation regarding the detrimental effect of glyphosate on glycemic health in humans, especially for those who have low circulating SHBG levels. Our findings might provide a novel basis for the understanding of the relationships between environmental glyphosate exposure, sex hormones, and development of glycemic disorder.

As one of the most widely used herbicides in the world, glyphosate is ubiquitous throughout the living and industrial environment and is becoming a critical threat to the public health [35]. Data from the Center for the Health Assessment of Mothers and Children of Salinas (CHAMACOS) showed that childhood exposure to glyphosate is associated with an increased risk of liver inflammation and metabolic syndrome in early adulthood [36]. A lipidomic analysis of human serum showed that glyphosate caused abnormal levels of several lipids, posing potential health risks [37]. The Agricultural Health Study (AHS) indicated that the use of glyphosate is significantly associated with incident hypothyroidism in private pesticide applicators [38]. However, the association between glyphosate and glycemic disorders remains largely unknown. Our findings showed positive associations of glyphosate withHbA1c and a negative association between glyphosate and HOMA-beta, results that agree with recent studies on glycemic disorders and glyphosate using the NHANES database [39,40,41]. Our results are also supported by the recent animal studies. For instance, in an in vivo and in silico analysis, the authors found that glyphosate exposure dose-dependently increased serum HbA1c levels and compromised pancreatic beta-cells in rats, which might cause one to speculate that glyphosate affects the expression of diabetes-related genes (*akt*, *irs-1*, *c-Src*, *β-arrestin-2*, *pi3k*, and *glut4*) via a direct molecular docking mechanism [42]. In addition, our RCS analysis highlighted non-monotonic inverted U-shaped associations between glyphosate and HbA1c in the general populations, and the inflection point was 0.50 ng/mL, respectively. It is noteworthy that these concentrations were approximate to the average level of glyphosate exposure of the populations included in this study (0.53 ng/mL) and were slightly higher than the exposure levels of general populations in California (GM = 0.28 ng/mL) [36]. These findings provided epidemiological clues and suggested the possible adverse effect of glyphosate on glycemic health in the low-dose and medium-dose environmental glyphosate exposure populations.

We further explored the effect of the interaction between sex hormones and glyphosate exposure on glycemic disorder, and the results showed that it was SHBG, rather than T or E2, that modified the association of glyphosate with glycemic disorder, including HOMA-IR, HOMA-IS, and FINS. SHBG is a plasma glycoprotein mainly secreted by hepatocytes. According to the “free hormone hypothesis”, SHBG regulates the free plasma concentration of T and E2 by binding to these circulating steroid hormones, thus regulating their bioavailability in target tissues and cells, a behavior that is believed to be the main role of SHBG [27]. However, the results of our study showed that T and E2 did not influence the association between glyphosate exposure and glycemic disorder, suggesting that SHBG may modulate glyphosate-induced glycemic disorder through a mechanism independent of T and E2. Moreover, many studies showed an inverse association between circulating SHBG levels and the risk of diabetes [43,44]. Specific single-nucleotide polymorphisms (SNPs) (such as rs6257, rs6259, and rs1799941) in the SHBG gene are risk factors for the development of diabetes [29,45,46]. The results of these genetic studies further demonstrate the causal direction of low SHBG, leading to glycemic disorder. The biological roles of SHBG are complex and not yet fully clear, and in this context, we propose some possible mechanisms by which SHBG modifies the association between glyphosate exposure and the increased risk of glycemic disorder through previous studies and the results of the present study. First, in vivo experiments have shown that glyphosate exposure leads to elevated H_2_O_2_ and LPO levels and reduced antioxidant levels, all of which affect membrane integrity and insulin receptor efficacy in the liver, leading to the development of insulin resistance and diabetes [19]. SHBG attenuates endoplasmic reticulum stress in hepatocytes both in vivo and in vitro [47], suggesting that high SHBG may be beneficial by reducing glyphosate-induced hepatocyte damage, thereby reducing the risk of glycemic disorder. Thus, individuals with low SHBG may be more sensitive to the increased risk of glycemic disorder from glyphosate. Second, glyphosate-based herbicide exposure in mice during puberty exacerbates high-fat diet-induced fat accumulation in adipocytes [48]. Excessive fat accumulation leads to the inflammation of adipose tissue, and obesity-associated chronic inflammation alters endocrine variables and interferes with insulin signaling pathways, all of which contribute to insulin resistance [49]. The addition of SHBG proteins protects equine adipose-derived stem/stromal cells from damaged and senescent phenotypes and inhibits adipogenesis [50]. Another study also showed that SHBG inhibits inflammation and lipid accumulation in macrophages and adipocytes, and the addition of testosterone or estradiol does not alter these effects of SHBG [51], which may suggest that high SHBG reduces the risk of glycemic disorder caused by glyphosate by inhibiting obesity-associated inflammation. These results collectively support the notion that SHBG might be an independent modulator in the glyphosate associated development of glycemic disorder. To this end, increased attention in the public health sector should be paid to the glycemic hazards of glyphosate on individuals with low SHBG levels.

This work possesses some notable advantages. First, the association between urinary glyphosate and glycemic outcomes was evaluated in non-institutionalized populations with high representativeness and large sample sizes, ensuring the generalizability of the findings. Second, we comprehensively explored the associations between urinary glyphosate exposure and glucose homeostasis and examined possible effect modifiers between the glyphosate-related associations, providing indispensable epidemiological evidence for the prevention of glycemic disorders in general populations, especially for vulnerable populations. Third, the levels of urinary glyphosate were measured using the advanced NHANES 2D-on-line IC-MS/MS method, and a series of glucose parameters were selected to assess the glycemic health of participants. However, some disadvantages should be acknowledged when interpreting the results. First, due to the cross-sectional nature of the NHANES, it was difficult to determine evidence of causality. Second, participants’ exposure to glyphosate was determined based on urinary glyphosate, and its major metabolite AMPA was not included in this study, as AMPA was not evaluated in the NHANES database. Third, our findings may be biased by potential confounders that could have been undetected or not noticed in this study. Fourth, the concentration of urinary glyphosate cannot represent the long-term exposure of participants due to the short half-life of glyphosate, and there may be other complex factors (genetic or biochemical factors) affecting urinary glyphosate concentration that have not been discovered at this stage.

## 5. Conclusions

In summary, we observed that exposure to glyphosate was associated with glycemic disorders, especially in those who had low serum SHBG levels. Low SHBG significantly aggravated the adverse associations between glyphosate and HOMA-IR, HOMA-IS, and FINS, a finding that offers a novel perspective regarding the roles of sex hormones in EDCs-induced glycemic disorders. More prospective cohort and experimental studies are needed to prove our findings.

## Figures and Tables

**Table 1:** General characteristics of the studied populations enrolled for glucose homeostasis analysis in NHANES 2013–2016.

	Glucose Homeostasis			
Demographic Characteristics	HOMA Parameters	FINS	HbA1c	FPG
*n*	1400	1403	2995	1428
HOMA-IR, mean (SD)	4.4 (8.5)			
HOMA-IS, mean (SD)	0.6 (0.5)			
HOMA-beta, mean (SD)	117.7 (138.2)			
FINS, mean (SD)		85.0 (119.9)		
HbA1c, mean (SD), %			5.8 (1.2)	
FPG, mean (SD), mmol/L				6.2 (2.1)
Age, mean (SD), years	50.19 (17.5)	50.2 (17.5)	49.4 (17.4)	50.2 (17.5)
Gender, *n* (%)				
Female	741 (52.9)	742 (52.9)	1558 (52.0)	759 (53.2)
Male	659 (47.1)	661 (47.1)	1437 (48.0)	669 (46.8)
Urinary creatinine, mean (SD), mg/dL	122.55 (76.69)	122.69 (76.70)	120.55 (78.33)	122.95 (77.18)
Glyphosate, mean (SD), ng/mL	0.489 (0.553)	0.489 (0.553)	0.527 (0.588)	0.492 (0.565)
Glyphosate, detection rate, %	71.89	71.96	73.08	71.70
Glyphosate, LLOD, ng/mL	0.200	0.200	0.200	0.200
TT, mean (SD), ng/dL	225.08 (252.08)	225.15 (251.94)	211.96 (235.79)	224.34 (251.91)
TT, detection rate, %	100.00	100.00	99.96	100.00
TT, LLOD, ng/mL	0.75	0.75	0.75	0.75
E2, mean (SD), pg/mL	64.17 (456.93)	64.06 (456.43)	58.09 (418.66)	64.20 (454.71)
E2, detection rate, %	94.83	94.84	94.28	94.80
E2, LLOD, pg/mL	2.99	2.99	2.99	2.99
SHBG, mean (SD), nmol/L	63.78 (58.74)	63.68 (58.71)	62.07 (52.60)	64.06 (58.91)
SHBG, detection rate, %	100.00	100.00	100.00	100.00
SHBG, LLOD, nmol/L	0.80	0.80	0.80	0.80
Ethnic, *n* (%)				
Mexican American	206 (14.7)	206 (14.7)	461 (15.4)	206 (14.4)
Non-Hispanic Black	256 (18.3)	257 (18.3)	583 (19.5)	264 (18.5)
Non-Hispanic White	595 (42.5)	597 (42.6)	1199 (40.0)	607 (42.5)
Other Hispanic	160 (11.4)	160 (11.4)	329 (11.0)	164 (11.5)
Other race—including multi-racial	183 (13.1)	183 (13.0)	423 (14.1)	187 (13.1)
PIR, *n* (%)				
No	1007 (78.2)	1010 (78.3)	2163 (78.6)	1027 (78.2)
Yes	280 (21.8)	280 (21.7)	588 (21.4)	286 (21.8)
Educational level, *n* (%)				
Less than 9th grade	130 (9.3)	130 (9.3)	254 (8.5)	131 (9.2)
9–11th grade (includes 12th grade with no diploma)	185 (13.2)	185 (13.2)	378 (12.6)	190 (13.3)
High-school graduate/GED or equivalent	284 (20.3)	284 (20.3)	662 (22.1)	290 (20.3)
Some college or AA degree	450 (32.2)	451 (32.2)	960 (32.1)	458 (32.1)
College graduate or above	349 (25.0)	351 (25.1)	737 (24.6)	357 (25.0)
BMI, *n* (%)				
<25	386 (27.7)	387 (27.7)	843 (28.3)	392 (27.6)
25–30	449 (32.2)	450 (32.2)	944 (31.7)	457 (32.1)
≥30	559 (40.1)	560 (40.1)	1190 (40.0)	573 (40.3)
Alcohol use, *n* (%)				
No	375 (28.5)	376 (28.5)	828 (29.6)	381 (28.4)
Yes	939 (71.5)	941 (71.5)	1970 (70.4)	960 (71.6)
Physical activity, *n* (%)				
No	841 (60.1)	842 (60.0)	1772 (59.2)	859 (60.2)
Moderate	281 (20.1)	282 (20.1)	602 (20.1)	286 (20.0)
Vigorous	278 (19.9)	279 (19.9)	621 (20.7)	283 (19.8)
Cigarette exposure, *n* (%)				
<LLOD	473 (33.8)	474 (33.8)	984 (33.0)	481 (33.8)
≥LLOD	927 (66.2)	929 (66.2)	1996 (67.0)	941 (66.2)
Cardiovascular disease, *n* (%)				
No	1244 (88.9)	1247 (88.9)	2663 (88.9)	1267 (88.7)
Yes	156 (11.1)	156 (11.1)	332 (11.1)	161 (11.3)
Stroke, *n* (%)				
No	1350 (96.5)	1353 (96.5)	2898 (96.9)	1378 (96.6)
Yes	49 (3.5)	49 (3.5)	94 (3.1)	49 (3.4)

Abbreviations: HOMA, homeostasis model assessment; HOMA-IR, homeostasis model assessment of insulin resistance; HOMA-IS, homeostasis model assessment of insulin sensitivity; HOMA-beta, homeostatic model assessment of beta-cell function; FINS, fasting serum insulin; HbA1c, hemoglobin A1c; FPG, fasting plasma glucose; SD, standard deviation; TT, total testosterone; E2, estradiol; SHBG, sex hormone-binding globulin; PIR, the ratio of family income to poverty; BMI, body mass index; LLOD, lower limit of detection.

**Table 2:** Effect of glyphosate, SHBG, and their interaction on glycemic outcomes in fully adjusted models.

	HOMA-IR	HOMA-IS	HOMA-Beta	FINS	HbA1c	FPG
Glyphosate	−0.04	0.04	−0.09	−0.06	0.01	0.02
	(−0.12, 0.04)	(−0.04, 0.12)	(−0.17, −0.01)	(−0.13, 0.01)	(0.01, 0.02)	(−0.00, 0.05)
	*p* = 0.349	*p* = 0.349	*p* = 0.024	*p* = 0.102	*p* = 0.001	*p* = 0.059
	*P*_FDR_ = 0.374	*P*_FDR_ = 0.374	*P*_FDR_ = 0.042	*P*_FDR_ = 0.147	*P*_FDR_ = 0.042	*P*_FDR_ = 0.077
SHBG	−0.24	0.24	−0.07	−0.19	−0.04	−0.05
	(−0.34, −0.14)	(0.14, 0.34)	(−0.15, 0.02)	(−0.28, −0.10)	(−0.05, −0.03)	(−0.08, −0.03)
	*p* < 0.001	*p* < 0.001	*p* = 0.142	*p* < 0.001	*p* < 0.001	*p* < 0.001
	*P*_FDR_ < 0.001	*P*_FDR_ < 0.001	*P*_FDR_ = 0.142	*P*_FDR_ < 0.001	*P*_FDR_ < 0.001	*P*_FDR_ < 0.001
Interaction	0.16	−0.16	0.09	0.13	0	0.03
	(0.02, 0.29)	(−0.29, −0.02)	(−0.04, 0.22)	(0.02, 0.24)	(−0.01, 0.02)	(−0.03, 0.08)
	*p* = 0.023	*p* = 0.023	*p* = 0.162	*p* = 0.024	*p* = 0.624	*p* = 0.308
	*P*_FDR_ = 0.051	*P*_FDR_ = 0.051	*P*_FDR_ = 0.227	*P*_FDR_ = 0.051	*P*_FDR_ = 0.624	*P*_FDR_ = 0.359

Abbreviations: HOMA-IR, homeostasis model assessment of insulin resistance; HOMA-IS, homeostasis model assessment of insulin sensitivity; HOMA-beta, homeostatic model assessment of beta-cell function; FINS, fasting insulin; HbA1c, glycated hemoglobin A1c; FPG, fasting plasma glucose. The association between glyphosate, SHBG and HOMA, FINS, HbA1c, and FPG is based on survey-weighted linear regression models, and the effect size is shown as *β* and 95% confidence interval. The model was adjusted for all covariates in this study.

**Table 3:** Subgroup analysis of the relationship between glyphosate and glycemic outcomes.

Outcome	Subgroup	*β* (95%CI)	*p*-Values	*P*_FDR_	*P* for Interaction	*P*_FDR_ for Interaction
HOMA-IR	SHBG				0.023	0.051
	High	−0.13 (−0.25, −0.02)	0.024	0.056		
	Low	0.07 (−0.05, 0.19)	0.231	0.323		
HOMA-IS	SHBG				0.023	0.051
	High	0.13 (0.02, 0.25)	0.024	0.056		
	Low	−0.07 (−0.19, 0.05)	0.231	0.323		
FINS	SHBG				0.024	0.051
	High	−0.14 (−0.24, −0.03)	0.013	0.056		
	Low	0.02 (−0.07, 0.11)	0.608	0.709		

Abbreviations: CI, confidence interval; SHBG, sex hormone-binding globulin; HOMA-IR, homeostasis model assessment of insulin resistance; HOMA-IS, homeostasis model assessment of insulin sensitivity; FINS, fasting insulin. Calculated via survey-weighted regression. The associations between glyphosate and HOMA, and FINS are based on linear regression models, and the effect size is shown as *β*. The model was adjusted for all covariates in this study.

## Data Availability

Publicly available datasets were analyzed in this study. These data can be found here: https://www.cdc.gov/nchs/nhanes/ (accessed on 2 June 2024).

## Supplementary Materials

The following supporting information can be downloaded at https://www.mdpi.com/article/10.3390/toxics12080600/s1, Table S1: Unadjusted analysis and adjusted analysis of the association between urinary glyphosate and glycemic outcomes in survey-weighted regression; Table S2: Association between urinary glyphosate and glycemic outcomes in unweighted regression; Table S3: Survey-weighted regression analysis of association between urinary glyphosate and glycemic outcomes in participants excluding missing data on covariates; Table S4: Statistic of interaction between covariates and urinary glyphosate on glycemic outcomes; Table S5: Mediation analysis of SHBG on the association between glyphosate and glycemic outcomes; Figure S1: Flowchart of population selection in NHANES 2013–2016; Figure S2: Proposed directed acyclic graph of the relation between urinary glyphosate and glucose homeostasis; Figure S3: Dose–effect relationships between urinary glyphosate and glycemic outcomes; Figure S4: The multiplicative interaction effects of SHBG on the association of glyphosate exposure and glycemic disorders.
