# Supplementary material for: Associations between Glyphosate Exposure and Glycemic Disorders: A Focus on the Modifying Effect of Sex Hormones
Source: Toxics. 2024 Aug 18;12(8):600. doi: 10.3390/toxics12080600 (PMC11359564; doi:10.3390/toxics12080600)
Supplement: Supplementary file 1 [file toxics-12-00600-s001.zip › toxics-3083963-supplementary.pdf]

Associations between glyphosate exposure and glycemic disorders: A focus on the modifying effect of sex hormones

Supplemental data

Table of Contents

Table S1. Unadjusted analysis and adjusted analysis of the association between urinary glyphosate and glycemic outcomes in survey-weighted regression.

Table S2. Association between urinary glyphosate and glycemic outcomes in unweighted regression.

Table S3. Survey-weighted regression analysis of association between urinary glyphosate and glycemic outcomes in participants excluding missing data on covariates.

Table S4. Statistic of interaction between covariates and urinary glyphosate on glycemic outcomes.

Table S5. Mediation analysis of SHBG on the association between glyphosate and glycemic outcomes.

Figure S1. Flowchart of population selection in NHANES 2013-2016.

Figure S2. Proposed directed acyclic graph for the relation between urinary glyphosate and glucose homeostasis.

Figure S3. Dose-effect relationships between urinary glyphosate and glycemic outcomes.

Figure S4. The multiplicative interaction effects of SHBG on the association of glyphosate exposure and glycemic disorders.

Table S1. Unadjusted analysis and adjusted analysis of the association between urinary glyphosate and glycemic outcomes in survey-weighted regression.

		Model 1			Model 2		
Outcome	n	β(95%CI)	*P* values	P_{FDR}	β (95%CI)	*P* values	P_{FDR}
HOMA-IR	1400	0.05(-0.03,0.13)	0.230	0.307	0.02(-0.06,0.10)	0.621	0.760
HOMA-IS	1400	-0.05(-0.13,0.03)	0.230	0.307	-0.02(-0.10,0.06)	0.621	0.760
HOMA-beta	1400	-0.08(-0.16,-0.01)	**0.032**	0.128	-0.06(-0.14,0.02)	0.151	0.378
FINS	1403	0.00(-0.07,0.08)	0.945	0.945	-0.01(-0.09,0.06)	0.760	0.760
HbA1c	2995	0.03(0.02,0.03)	**<0.001**	**<0.001**	0.02(0.01,0.02)	**<0.001**	**<0.001**
FPG	1428	0.05(0.02,0.07)	**<0.001**	**<0.001**	0.03(0.01,0.05)	**0.015**	0.075

Abbreviations: CI, confidence interval; HOMA-IR, homeostasis model assessment of insulin resistance; HOMA-IS, homeostasis model assessment of insulin sensitivity; HOMA-beta, homeostatic model assessment of beta-cell function; FINS, fasting serum insulin; HbA1c, hemoglobin A1c; FPG, Fasting plasma glucose.

he association between glyphosate and HOMA, FINS, HbA1c, and FPG was estimated using linear regression models, and the effect size was shown as β.

The model 1 was crude model.

The model 2 was adjusted for urinary creatinine, age, gender, ethnicity, the ratio of family income to poverty, education level.

Table S2. Association between urinary glyphosate and glycemic outcomes in unweighted regression.

Outcome	n	β (95%CI)	*P* values	P_{FDR}
HOMA-IR	1400	-0.04(-0.12,0.04)	0.344	0.344
HOMA-IS	1400	0.04(-0.04,0.12)	0.344	0.344
HOMA-beta	1400	-0.09(-0.17,-0.01)	**0.023**	0.054
FINS	1403	-0.06(-0.13,0.01)	0.106	0.148
HbA1c	2995	0.01(0.01,0.02)	**0.001**	**0.004**
FPG	1428	0.02(-0.00,0.05)	0.058	0.102

Abbreviations: CI, confidence intervals; HOMA-IR, homeostasis model assessment of insulin resistance; HOMA-IS, homeostasis model assessment of insulin sensitivity; HOMA-beta, homeostatic model assessment of beta-cell function; FINS, fasting serum insulin; HbA1c, hemoglobin A1c; FPG, Fasting plasma glucose.

The association between glyphosate and HOMA, FINS, HbA1c, and FPG was based on linear regression models, and the effect size was shown as β.

The model was adjusted for all covariates in this study (urinary creatinine, age, gender, ethnicity, educational level, family income to poverty ratio, cigarette exposure, alcohol consumption, physical activity, body mass index, cardiovascular disease, stroke and urinary creatinine levels).

Table S3. Survey-weighted regression analysis of association between urinary glyphosate and glycemic outcomes in participants excluding missing data on covariates.

Outcome	n	β (95%CI)	*P* values	P_{FDR}
HOMA-IR	1062	-0.04(-0.13,0.05)	0.374	0.374
HOMA-IS	1062	0.04(-0.05,0.13)	0.374	0.374
HOMA-beta	1062	-0.11(-0.19,-0.03)	**0.012**	**0.042**
FINS	1065	-0.07(-0.15,0.02)	0.105	0.147
HbA1c	2246	0.01(0.00,0.02)	**0.008**	**0.042**
FPG	1073	0.03(0.00,0.05)	**0.044**	0.077

Abbreviations: CI, confidence interval; HOMA-IR, homeostasis model assessment of insulin resistance; HOMA-IS, homeostasis model assessment of insulin sensitivity; HOMA-beta, homeostatic model assessment of beta-cell function; FINS, fasting serum insulin; HbA1c, hemoglobin A1c; FPG, Fasting plasma glucose.

The association between glyphosate and HOMA, FINS, HbA1c, and FPG was based on linear regression models, and the effect size was shown as β.

The model was adjusted for all covariates in this study (urinary creatinine, age, gender, ethnicity, educational level, family income to poverty ratio, cigarette exposure, alcohol consumption, physical activity, body mass index, cardiovascular disease, stroke and urinary creatinine levels).

Table S4. Statistic of interaction between covariates and urinary glyphosate on glycemic outcomes.

	**HOMA-IR**	**HOMA-IS**	**HOMA-beta**	**FINS**	**HbA1c**	**FPG**
Gender	0.00 (-0.11,0.11) $P = 0.978$, $P_{FDR} = 0.992$	-0.00 (-0.11,0.11) $P = 0.978$, $P_{FDR} = 0.992$	-0.00 (-0.13,0.13) $P = 0.992$, $P_{FDR} = 0.992$	-0.01 (-0.12,0.10) $P = 0.891$, $P_{FDR} = 0.992$	-0.00 (-0.01,0.01) $P = 0.691$, $P_{FDR} = 0.992$	0.01 (-0.02,0.04) $P = 0.578$, $P_{FDR} = 0.992$
Ethnic	-0.10 (-0.28,0.08) $P = 0.270$, $P_{FDR} = 0.474$	0.10 (-0.08,0.28) $P = 0.270$, $P_{FDR} = 0.474$	0.03 (-0.12,0.18) $P = 0.665$, $P_{FDR} = 0.665$	-0.06 (-0.22,0.09) $P = 0.413$, $P_{FDR} = 0.503$	0.01 (-0.02,0.03) $P = 0.431$, $P_{FDR} = 0.503$	-0.02 (-0.07,0.02) $P = 0.271$, $P_{FDR} = 0.474$
Educational level	-0.10 (-0.36,0.16) $P = 0.453$, $P_{FDR} = 0.640$	0.10 (-0.16,0.36) $P = 0.453$, $P_{FDR} = 0.640$	-0.06 (-0.23,0.11) $P = 0.457$, $P_{FDR} = 0.640$	-0.09 (-0.31,0.13) $P = 0.408$, $P_{FDR} = 0.640$	0.00 (-0.03,0.03*)* $P = 0.855$, $P_{FDR} = 0.855$	-0.02 (-0.08,0.05) $P = 0.622$, $P_{FDR} = 0.726$
Cigarette exposure	-0.05 (-0.17,0.06) $P = 0.375$, $P_{FDR} = 0.656$	0.05 (-0.06,0.17) $P = 0.375$, $P_{FDR} = 0.656$	-0.01 (-0.13,0.11) $P = 0.833$, $P_{FDR} = 0.863$	-0.04 (-0.14,0.07) $P = 0.507$, $P_{FDR} = 0.710$	-0.01 (-0.03,0.01) $P = 0.247$, $P_{FDR} = 0.656$	-0.02 (-0.05,0.01) $P = 0.266$, $P_{FDR} = 0.656$
Alcohol use	-0.01 (-0.17,0.15) $P = 0.903$, $P_{FDR} = 0.988$	0.01 (-0.15,0.17) $P = 0.903$, $P_{FDR} = 0.988$	0.04 (-0.09,0.17) $P = 0.550$, $P_{FDR} = 0.988$	0.00 (-0.15,0.15) $P = 0.988$, $P_{FDR} = 0.988$	0.01 (-0.01,0.03) $P = 0.592$, $P_{FDR} = 0.988$	-0.02 (-0.05,0.01) $P = 0.130$, $P_{FDR} = 0.910$
Physical activity	-0.05 (-0.17,0.07) $P = 0.393$, $P_{FDR} = 0.458$	0.05 (-0.07,0.17) $P = 0.393$, $P_{FDR} = 0.458$	0.12 (-0.08,0.32) $P = 0.218$, $P_{FDR} = 0.458$	0.01 (-0.12,0.13) $P = 0.927$, $P_{FDR} = 0.927$	-0.01 (-0.03,0.01) $P = 0.325$, $P_{FDR} = 0.458$	-0.05 (-0.11,0.01) $P = 0.089$, $P_{FDR} = 0.458$
BMI	-0.02 (-0.17,0.12) $P = 0.726$, $P_{FDR} = 0.847$	0.02 (-0.12,0.17) $P = 0.726$, $P_{FDR} = 0.847$	-0.04 (-0.19,0.11) $P = 0.566$, $P_{FDR} = 0.847$	-0.04 (-0.17,0.10) $P = 0.594$, $P_{FDR} = 0.847$	-0.00 (-0.01,0.01*)* $P = 0.890$, $P_{FDR} = 0.890$	0.01 (-0.02,0.04) $P = 0.602$, $P_{FDR} = 0.847$
TT	-0.00 (-0.13,0.13) $P = 0.949$, $P_{FDR} = 0.954$	0.00 (-0.13,0.13) $P = 0.949$, $P_{FDR} = 0.954$	0.01 (-0.13,0.15) $P = 0.865$, $P_{FDR} = 0.954$	0.01 (-0.12,0.14) $P = 0.877$, $P_{FDR} = 0.954$	0.00 (-0.01,0.01) $P = 0.914$, $P_{FDR} = 0.954$	-0.01 (-0.04,0.02) $P = 0.419$, $P_{FDR} = 0.954$
E2	-0.10 (-0.24,0.04) $P = 0.142$, $P_{FDR} = 0.320$	0.10 (-0.04,0.24) $P = 0.142$, $P_{FDR} = 0.320$	0.00 (-0.14,0.14) $P = 0.985$, $P_{FDR} = 0.985$	-0.07 (-0.19,0.06) $P = 0.286$, $P_{FDR} = 0.400$	-0.01 (-0.02,0.01) $P = 0.520$, $P_{FDR} = 0.607$	-0.04 (-0.07,0.00) $P = 0.079$, $P_{FDR} = 0.320$

Abbreviations: TT, total testosterone; E2, estradiol; PIR, the ratio of family income to poverty; BMI, body mass index; HOMA-IR, homeostasis model assessment of insulin resistance; HOMA-IS, homeostasis model assessment of insulin sensitivity; HOMA-beta, homeostatic model assessment of beta-cell function; FINS, fasting insulin; HbA1c, glycated hemoglobin A1c; FPG, fasting plasma glucose. *: *P for interaction* < 0.05.

Table S5. Mediation analysis of SHBG on the association between glyphosate and glycemic outcomes.

Mediator	Outcome	Total effect	Direct effect	Indirect effect	Proportion
SHBG	HOMA-IR	0.015 (-0.060,0.090)	0.015 (-0.054,0.077)	0.001 (-0.024,0.023)	10.9%
SHBG	HOMA-IS	-0.017 (-0.091,0.041)	-0.017 (-0.085,0.046)	-0.000 (-0.023,0.026)	9.8%
SHBG	FINS	-0.013 (-0.080,0.053)	-0.013 (-0.075,0.043)	0.000 (-0.020,0.019)	14.1%

Abbreviations: HOMA-IR, homeostasis model assessment of insulin resistance; HOMA-IS, homeostasis model assessment of insulin sensitivity; FINS, fasting serum insulin; SHBG, sex hormone binding globulin.

The model was adjusted for all covariates in this study (urinary creatinine, age, gender, ethnicity, educational level, family income to poverty ratio, cigarette exposure, alcohol consumption, physical activity, body mass index, cardiovascular disease, stroke and urinary creatinine levels).

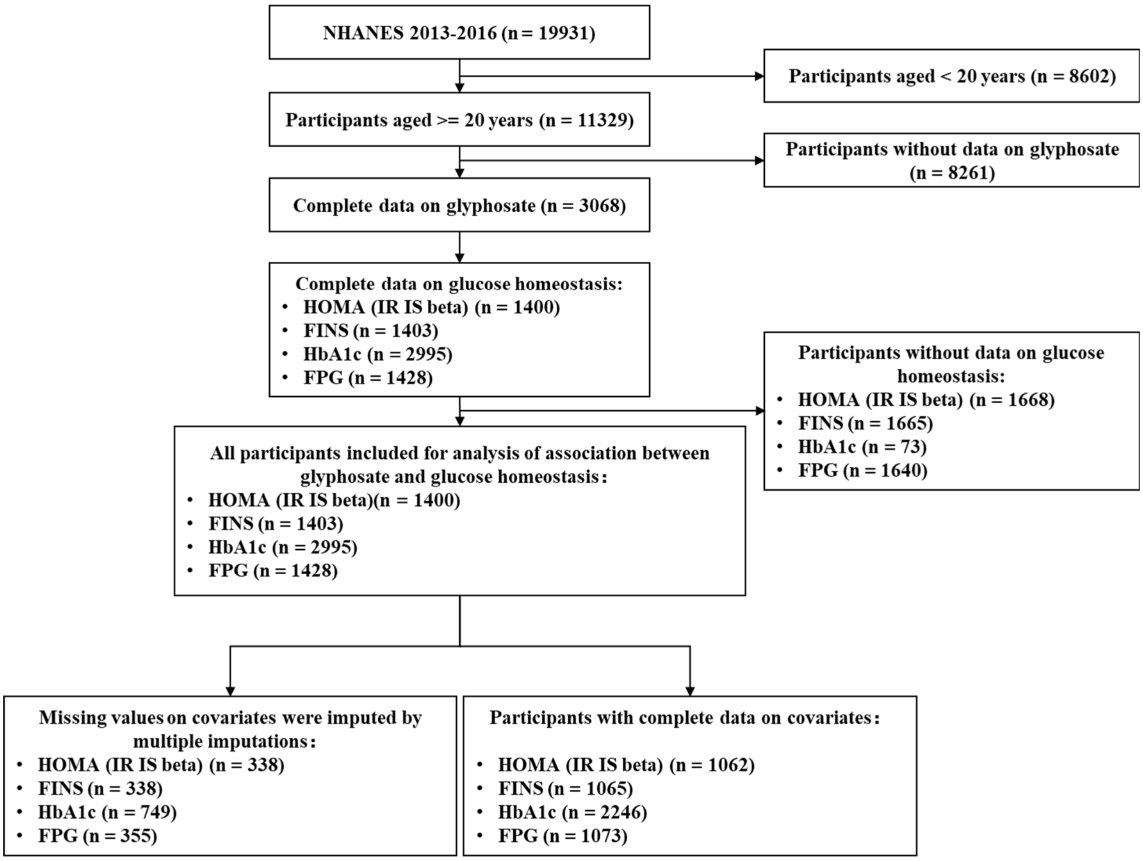

Figure S1. Flowchart of population selection in NHANES 2013-2016.

Abbreviations: HOMA-IR, homeostasis model assessment of insulin resistance; HOMA-IS, homeostasis model assessment of insulin sensitivity; HOMA-beta, homeostatic model assessment of beta-cell function; FINS, fasting insulin; HbA1c, glycated hemoglobin A1c; FPG, fasting plasma glucose.

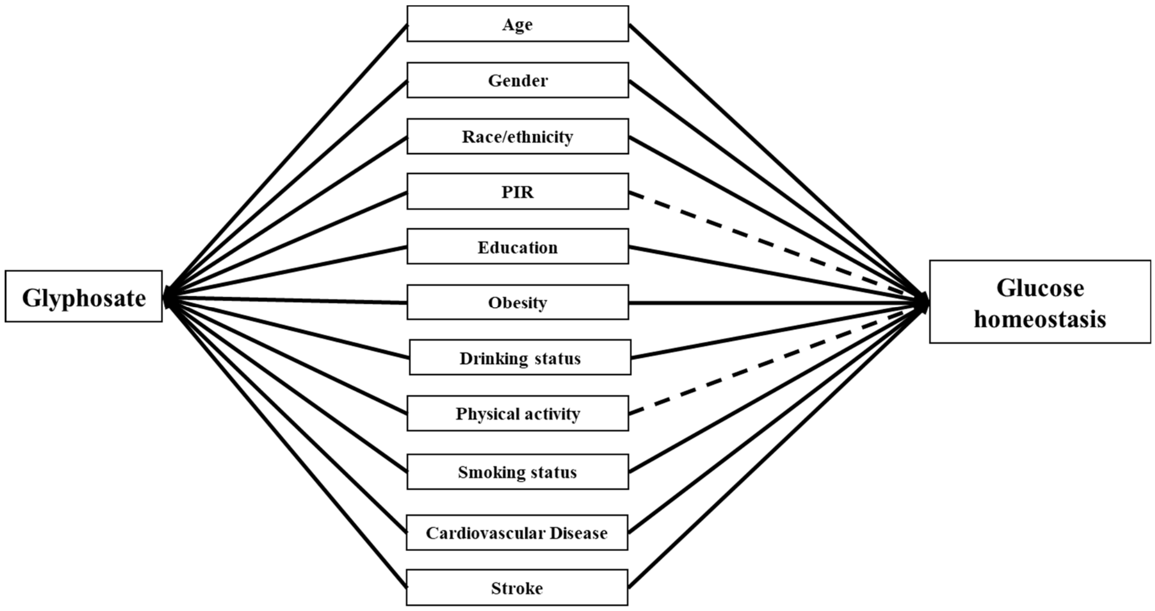

Figure S2. Proposed directed acyclic graph for the relation between urinary glyphosate and glucose homeostasis.

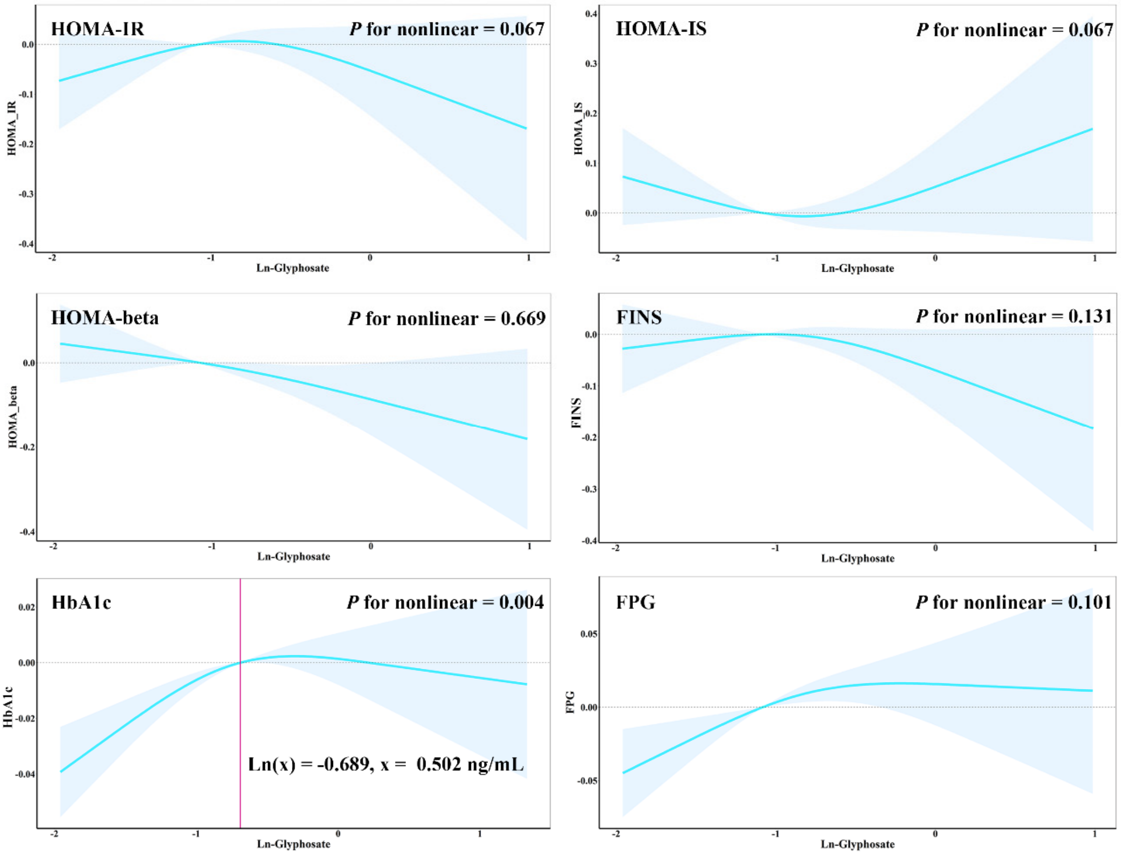

Figure S3. Dose-effect relationships between urinary glyphosate and glycemic outcomes.

The regression models were adjusted by age, gender, ethnicity, educational level, family income to poverty ratio, cigarette exposure, alcohol consumption, physical activity, body mass index, cardiovascular disease, stroke and urinary creatinine levels. Abbreviations: HOMA-IR, homeostasis model assessment of insulin resistance; HOMA-IS, homeostasis model assessment of insulin sensitivity; HOMA-beta, homeostatic model assessment of beta-cell function; FINS, fasting insulin; HbA1c, glycated hemoglobin A1c; FPG, fasting plasma glucose.

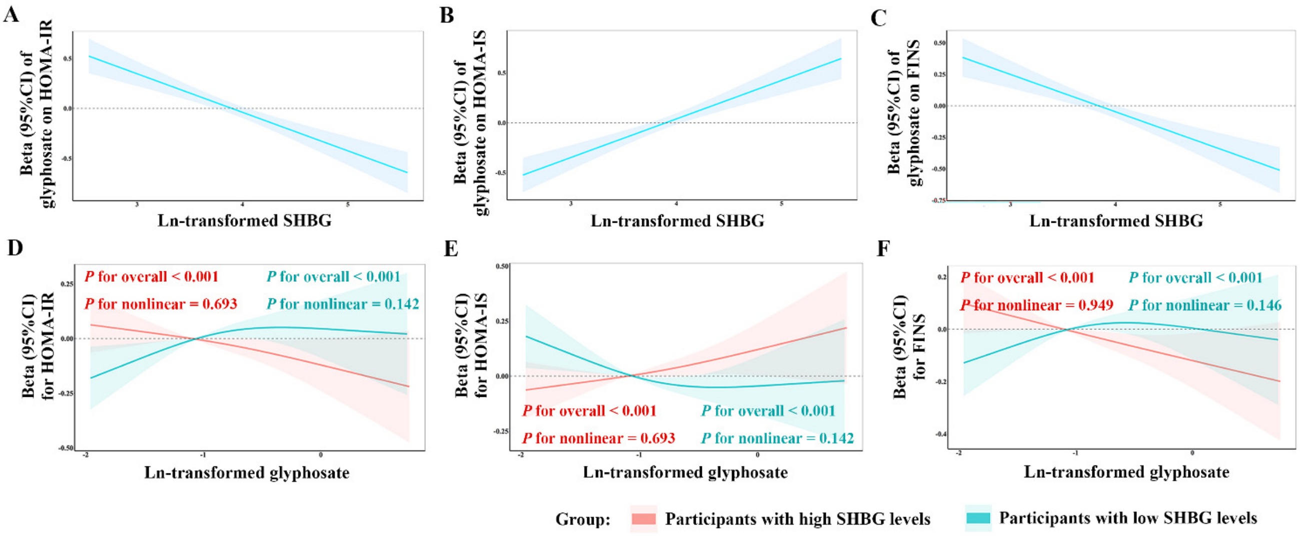

Figure S4. The multiplicative interaction effects of SHBG on the association of glyphosate exposure and glycemic disorders.

The effect of interaction between urinary glyphosate and SHBG on elevated HOMA-IR (A), HOMA-IS (B), and FINS (C). Association of urinary glyphosate with HOMA-IR (D), HOMA-IS (E), and FINS (F) according to SHBG. The models were adjusted by age, gender, ethnicity, educational level, family income to poverty ratio, cigarette exposure, alcohol consumption, physical activity, body mass index, cardiovascular disease, stroke and urinary creatinine levels. Abbreviations: CI, confidence interval; SHBG, sex hormone binding globulin; HOMA-IR, homeo-stasis model assessment of insulin resistance; HOMA-IS, homeostasis model assessment of insulin sensitivity; FINS, fasting insulin.
